# Efficacy and Safety of Antidepressants for the Treatment of Irritable Bowel Syndrome: A Meta-Analysis

**DOI:** 10.1371/journal.pone.0127815

**Published:** 2015-08-07

**Authors:** Chen Xie, Yurong Tang, Yunfeng Wang, Ting Yu, Yun Wang, Liuqin Jiang, Lin Lin

**Affiliations:** 1 Department of Gastroenterology, the First Affiliated Hospital Nanjing Medical University, Nanjing, China; 2 Library of Nanjing Medical University, Nanjing, China; Peking University, CHINA

## Abstract

**Aim:**

The aim of this meta-analysis was to analyze the efficacy and safety of antidepressants for the treatment of irritable bowel syndrome.

**Methods:**

We searched MEDLINE, EMBASE, Scopus and The Cochrane Library for randomized controlled trials investigating the efficacy and safety of antidepressants in the treatment of irritable bowel syndrome. Article quality was evaluated by Jadad score. RevMan 5.0 and Stata 12.0 were used for the meta-analysis.

**Results:**

Twelve randomized controlled trials were included in this study and most of these trials were of high quality (Jadad score ≥4). Five articles focused on tricyclic antidepressants, six articles involved selective serotonin reuptake inhibitors, and one article investigated both types of treatment. The pooled risk ratio showed antidepressant treatment can improve global symptoms (RR = 1.38, 95% CI 1.08, 1.77). In the subgroup analysis, treatment with tricyclic antidepressants showed an improvement in global symptoms (RR = 1.36, 95% CI 1.07, 1.71), while treatment with selective serotonin reuptake inhibitors showed no statistically significant difference in global symptoms compared with the control groups (RR = 1.38, 95% CI 0.83, 2.28). The pooled risk ratio of dropout due to side effects following antidepressant treatment was 1.71 with 95% CI (0.98, 2.99). The subgroup analysis showed the pooled risk ratio of dropout in the tricyclic antidepressants group was 1.92 with 95% CI (0.89, 4.17). In the selective serotonin reuptake inhibitors group, the pooled risk ratio of dropout was 1.5 with 95% CI (0.67, 3.37). Selective serotonin reuptake inhibitors showed no benefit in alleviating abdominal pain and improving quality of life. There was no difference in the incidence of common adverse events between treatment and control groups.

**Conclusions:**

TCAs can improve global symptoms of irritable bowel syndrome, while there was no strong evidence to confirm the effectiveness of SSRIs for the treatment of IBS.

## Introduction

Irritable bowel syndrome (IBS) is one of the most common bowel diseases, which seriously affects the quality of life of the patient and consumes a considerable amount of medical resources [1]. To date, there is no universally accepted method to effectively cure this disease. Most popular medicines, including antispasmodics, antidiarrheals, and laxatives, only treat the symptoms of IBS and are therefore not ideal.

A substantial number of studies indicated that IBS patients have abnormal personality with higher anxiety-depression scores [2–4]. Hence, several studies were conducted to evaluate the effectiveness of antidepressants on IBS. The most commonly used antidepressants in the treatment of IBS are tricyclic antidepressants (TCAs; e.g., imipramine, desipramine, and amitriptyline) and selective serotonin reuptake inhibitors (SSRIs; e.g., fluoxetine, paroxetine, and citalopram). Although these antidepressants have been used in IBS treatment, the clinical evidence of their efficacy is still controversial.

Recently, a meta-analysis regarding the effectiveness of antidepressants in IBS treatment was published [5]. However, one of the references listed in this article contained inconsistent data [6] and this meta-analysis did not adopt fixed assessment criteria. Besides, previously published meta-analysis rarely discussed any of the adverse effects associated with the use of antidepressants. More importantly is that several new studies have been published in recent years. In order to obtain more accurate and comprehensive results, we decided to conduct this meta-analysis to evaluate the efficacy and safety of antidepressants for the treatment of IBS.

## Methods

### Search strategy

A literature search was conducted on MEDLINE, EMBASE, Scopus and The Cochrane Library. References on identified articles were also reviewed for additional articles missed by the computerized database search. Data published between 1966 and September 2014 were collected. In this study the following terms were used to identify IBS: functional gastrointestinal disorder, refractory irritable bowel symptoms, irritable bowel syndrome or IBS. These terms were combined using the set operator “AND” with: antidepressants, anxiolytics, antipsychotics, hypnosedatives, tricyclic antidepressants, selective serotonin reuptake inhibitors, serotonin-norepinephrine reuptake inhibitors, atypical antipsychotics, imipramine, desipramine, amitriptyline, doxepin, clomipramine, maprotiline, nortriptyline, fluoxetine, paroxetine, sertraline, tianeptine, citalopram, trazodone, mianserin, mirtazapine, and venlafaxine.

### Inclusion and exclusion criteria

Inclusion criteria: (1) IBS was definitively diagnosed by clinical diagnosis or by Rome I, II, or III criteria. (2) Age above 18 years old. (3) Treatment groups used antidepressants, while control groups used placebo or usual therapy. (4) To avoid carry-over effects, we only included cross-over studies that provided outcome data from the first period. (5) The duration of the treatment and follow-up was 7 days at least for all groups.

Exclusion criteria: (1) Studies did not distinguish IBS from functional gastrointestinal disorder. (2) Age below 18 years old. (3) Treatment groups did not use antidepressants or combine different antidepressants in one patient. (4) No control group. (5) Not a randomized controlled trial (RCT). (6) Unable to extract data from original literature. (7) Cross-over studies did not provide outcome data of the first period. (8) Duplicate publication. (9) No full text was available. (10) Language was not English.

### Outcomes

One of the primary measurements was the proportion of patients with global symptom relief. Another primary attribute was the rate of dropout due to side effects. Secondary outcomes included the degree of improvement in both abdominal pain and in quality of life. We also analyzed the pooled risk ratio (RR) of the incidence of common adverse events.

### Literature quality evaluation

We used the Jadad score to evaluate the quality of eligible articles. Jadad score evaluates literature quality by analyzing the generation of random sequences, randomization concealment, blinding, and dropouts. Articles with a Jadad score between one and three were considered to be low quality studies, while articles with a score between four and seven were deemed to be high quality studies.

### Data extraction and analysis

Original data were extracted and presented in a four-fold table. Then all data were analyzed with RevMan 5.0 and Stata 12.0. RevMan 5.0 was used to calculate the pooled effect size and Stata 12.0 was used to assess publication bias and for sensitivity analysis.

Dichotomous data were analyzed by RR and 95% CI. Continuous variable data were analyzed by standardized mean difference. In order to gain more conservative results, the analysis was on an intention-to-treat (ITT) basis, regardless of whether or not the original authors had performed such an analysis. Dropouts or withdrawals before the completion of the studies were considered as treatment failures.

The I^2^ index was used to qualitatively analyze heterogeneity. If I^2^ was <25%, this indicated there was low heterogeneity and a fixed effect model was applied to pool effect size. An I^2^ value >25% and <50% suggested there was moderate heterogeneity. If I^2^ was >50%, it showed there was significant heterogeneity [7]. In order to achieve more conservative results, a random effect model was applied to pool effect size in the last two conditions above [8]. Sensitivity analysis was conducted when heterogeneity was significant. This method allowed us to find the source of heterogeneity and to judge the robustness of the results.

Publication bias was assessed qualitatively by funnel plot and quantitatively by Begg’s test. We considered there was no publication bias if the funnel plot was symmetrical and the P-value was >0.05.

## Results

**Fig 1:**
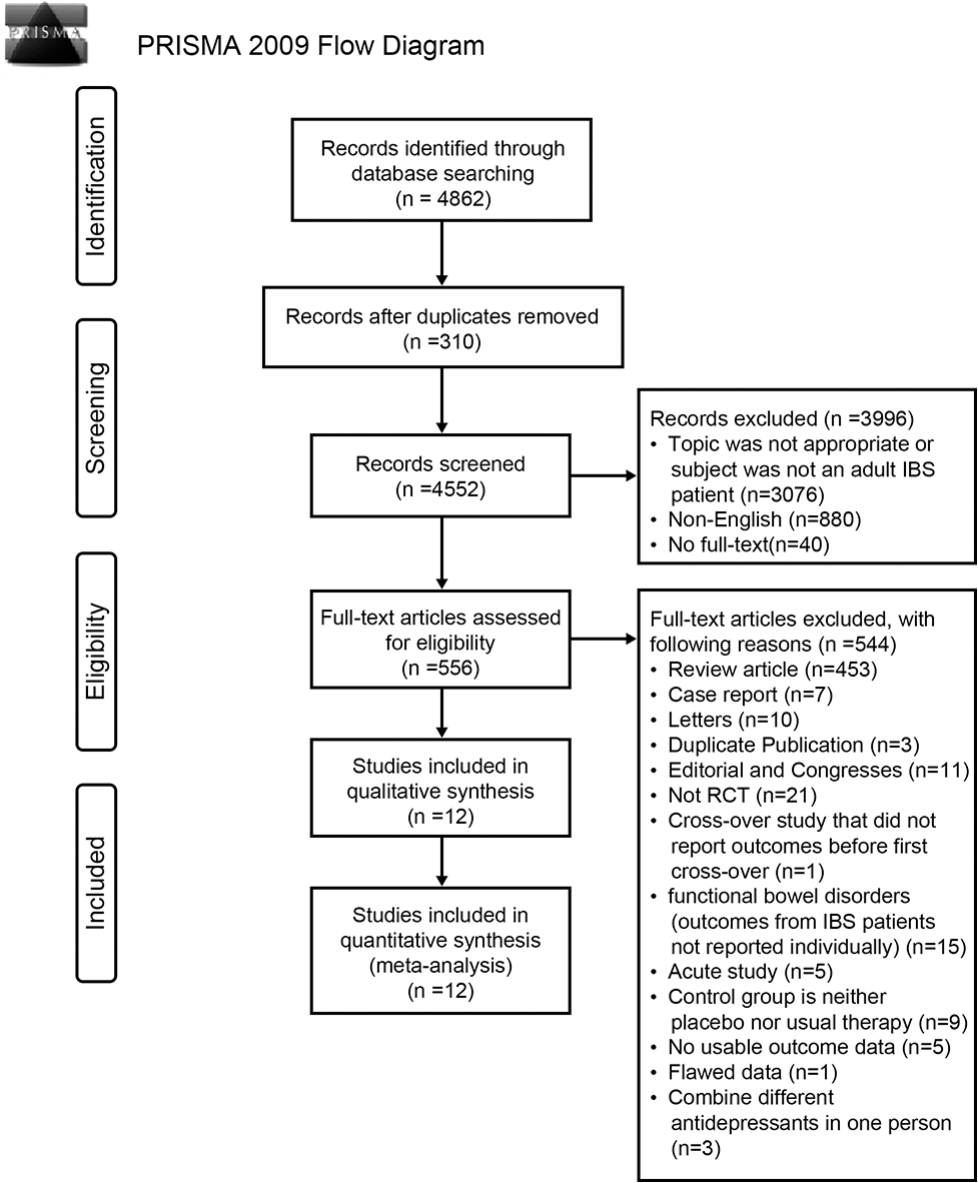
Detailed screening process.

The literature search identified 4862 studies. Based on the inclusion and exclusion criteria, 12 RCTs were eligible for inclusion in this study. The detailed screening process is shown in Fig 1.

Among the included studies, five articles focused on TCAs [9–13], six articles involved SSRIs [14–19], and one article investigated both TCAs and SSRIs [20]. There were 10 high quality studies (Jadad score ≥4). The characteristics of studies included in the meta-analysis are listed in Table 1.

**Table 1:** Characteristics of the studies included in the meta-analysis.

Study (First Author)	Country	Year of publication	Jadad Score	Diagnostic Criteria	Type	Depression Comorbidity	Control	Sample (M/F)	No. of subjects in treatment group	Dropout due to side effects in treatment group	No. of subjects in control group	Dropout due to side effects in control group	Antidepressants	Course	Follow-up
**Heefner JD**	**USA**	**1978**	**4**	**clinical diagnosis and investigations**	**no report**	**few**	**placebo**	**44(no report)**	**22**	**3**	**22**	**1**	**Desipramine 150mg qn**	**2M**	**2M**
**Myren J**	**Norway**	**1982**	**3**	**clinical diagnosis and investigations**	**no report**	**no report**	**placebo**	**61(28/33)**	**30**	**0**	**31**	**0**	**Trimipramine 50mg qn**	**4W**	**4W**
**Drossman**	**USA and Canada**	**2003**	**7**	**Rome I**	**no report**	**partly**	**placebo**	**172(0/172)**	**115**	**/**	**57**	**/**	**Desipramine 50 mg qdfor 1 week, 100 mg qd for 1 week, and then 150 mg qd from week 3 to week 12**	**12W**	**12W**
**Vahedi H**	**Iran**	**2008**	**6**	**Rome II**	**IBS-D**	**no report**	**placebo**	**54(30/24)**	**27**	**1**	**27**	**1**	**Amitriptyline 10 mg qn**	**2M**	**2M**
**Abdul-Baki H**	**Lebanon**	**2009**	**7**	**Rome II**	**IBS-C, IBS-D, IBS-M**	**no**	**placebo**	**107(62/45)**	**59**	**14**	**48**	**6**	**Imipramine 25mg qd. Doubling the dose was allowed once at week 2.**	**12W**	**12W**
**Masand PS**	**USA**	**2009**	**4**	**Rome II**	**no report**	**no**	**placebo**	**72(9/63)**	**36**	**3**	**36**	**2**	**Paroxetine controlled-release;started at 12.5mg po qd,increased biweekly in 12.5 mg/d; maximum dose 50mg po qd; mean dose 30mg po qd**	**12 W**	**12 W**
**Creed F**	**England**	**2003**	**3**	**Rome I**	**IBS-C, IBS-D, other**	**partly**	**routine care**	**172(35/137)**	**86**	**29**	**86**	**/**	**Paroxetine 20 mg po qd**	**3 M**	**3M, 1Y**
**Kuiken SD**	**Holland**	**2003**	**7**	**Rome I**	**IBS-C, IBS-D, IBS-A**	**no**	**placebo**	**40(18/22)**	**19**	**2**	**21**	**4**	**Fluoxetine 20mg po qn**	**6 W**	**6 W**
**Tack J**	**Belgium**	**2006**	**4**	**RomeII**	**IBS-C, IBS-D, IBS-A**	**no**	**placebo**	**23(5/18)**	**11**	**1**	**12**	**1**	**Citalopram 20 mg during the first three weeks and 40 mg during the second three weeks**	**6 W**	**3 W, 6W**
**Ladabaum U**	**USA**	**2010**	**7**	**Rome II**	**IBS-C, IBS-D, IBS-A**	**no**	**placebo**	**54(10/44)**	**27**	**7**	**27**	**2**	**Citalopram 20 mg qd for 4 weeks, then 40 mg qd for 4 weeks**	**8 W**	**4 W, 8 W**
**Vahedi H**	**Iran**	**2005**	**6**	**Rome II**	**pain and IBS-C**	**no severe**	**placebo**	**44(17/27)**	**22**	**0**	**22**	**0**	**Fluoxetine 20mg po qd**	**12W,16W**	**12W, 16W**
**Talley NJ**	**Australia**	**2008**	**6**	**Rome II**	**IBS-C, IBS-D**	**no**	**placebo**	**34(13/21)**	**18**	**/**	**16**	**/**	**Imipramine beginning at a dose of 25 mg qn; then increased 2 weeks later to 50 mg qn**	**12W**	**12W**
**Talley NJ**	**Australia**	**2008**	**6**	**Rome II**	**IBS-C, IBS-D**	**no**	**placebo**	**33(13/20)**	**17**	**/**	**16**	**/**	**Citalopram beginning at 20 mg qd andincreased to 40 mg qd 2 weeks later**	**12W**	**12W**

Nine articles evaluated the effects of antidepressants on global symptom relief [10–16, 18, 20]: four studies assessed TCAs [10–13], four studies investigated SSRIs [14–16, 18], and one study compared TCAs, SSRIs, and placebo at the same time [20]. The definitive criteria for calculating the percentage of patients with global symptom relief and the result in each included study can be found in the Table 2.

**Table 2:** Details of the calculations for the proportion of global symptom relief.

Antidepressants	Study(First Author and year of publication)	The definition criteria for the calculation of proportion of global symptom relief				Treatment Group	Treatment Group	Control Group	Control Group
						Improved	Total	Improved	Total
**TCAs**	**Myren J 1982**	**A feeling of well-being. After therapy, IBS patients were divided into three groups (ie. improved, unchanged and worse) and the result was calculated as the proportion of the improved group.**				**25**	**30**	**21**	**31**
	**Drossman 2003**	**Global well-being (“how would you rate your general well-being”) scored on a Likert scale from 1 (poor) to 5 (excellent) and was calculated according to the proportion of “good, very good and excellent”.**				**55**	**115**	**21**	**57**
	**Vahedi 2008**	**Calculated the proportion of the absence of all symptoms.**				**17**	**27**	**7**	**27**
	**Talley NJ 2008**	**Calculated the proportion of “adequate relief over 50% in a week”.**				**10**	**18**	**9**	**16**
	**Abdul-Baki H 2009**	**Assessed by asking the following question: “Have your symptoms improved satisfactorily since starting the drug study?” The result was calculated as per to the proportion of the improved group.**				**25**	**59**	**12**	**48**
**SSRIs**	**Kuiken SD 2003**	**Assessed by asking the following question: “Please consider how you felt during the past 2 weeks in regard to your irritable bowel syndrome. Compared to the way you felt before entering the study, how would you rate your relief of symptoms during the past 2 weeks?”. Possible answers were relieved, unchanged, or worse. The result was calculated as per to the proportion of the relieved group**				**10**	**19**	**9**	**21**
	**Creed F 2003**	**After therapy, IBS patients were divided into three groups (ie. improved, same and worse) and the result was calculated as per to the proportion of the improved group**				**49**	**86**	**30**	**86**
	**Talley NJ 2008**	**Calculated the proportion of “adequate relief over 50% in a week”**				**8**	**17**	**9**	**16**
	**Masand PS 2009**	**The responders were defined as subjects with clinical global impression–improvement (CGI–I) scores of 1 or 2 at the end of treatment The result was calculated as per to the proportion of the responders.**				**25**	**36**	**6**	**36**
	**Ladabaum U 2010**	**Overall response was defined as achieving self-reported weekly “adequate relief” for at least three of the last 6 weeks. The result was calculated as per to the proportion of the responders.**				**12**	**27**	**15**	**27**

**Fig 2:**
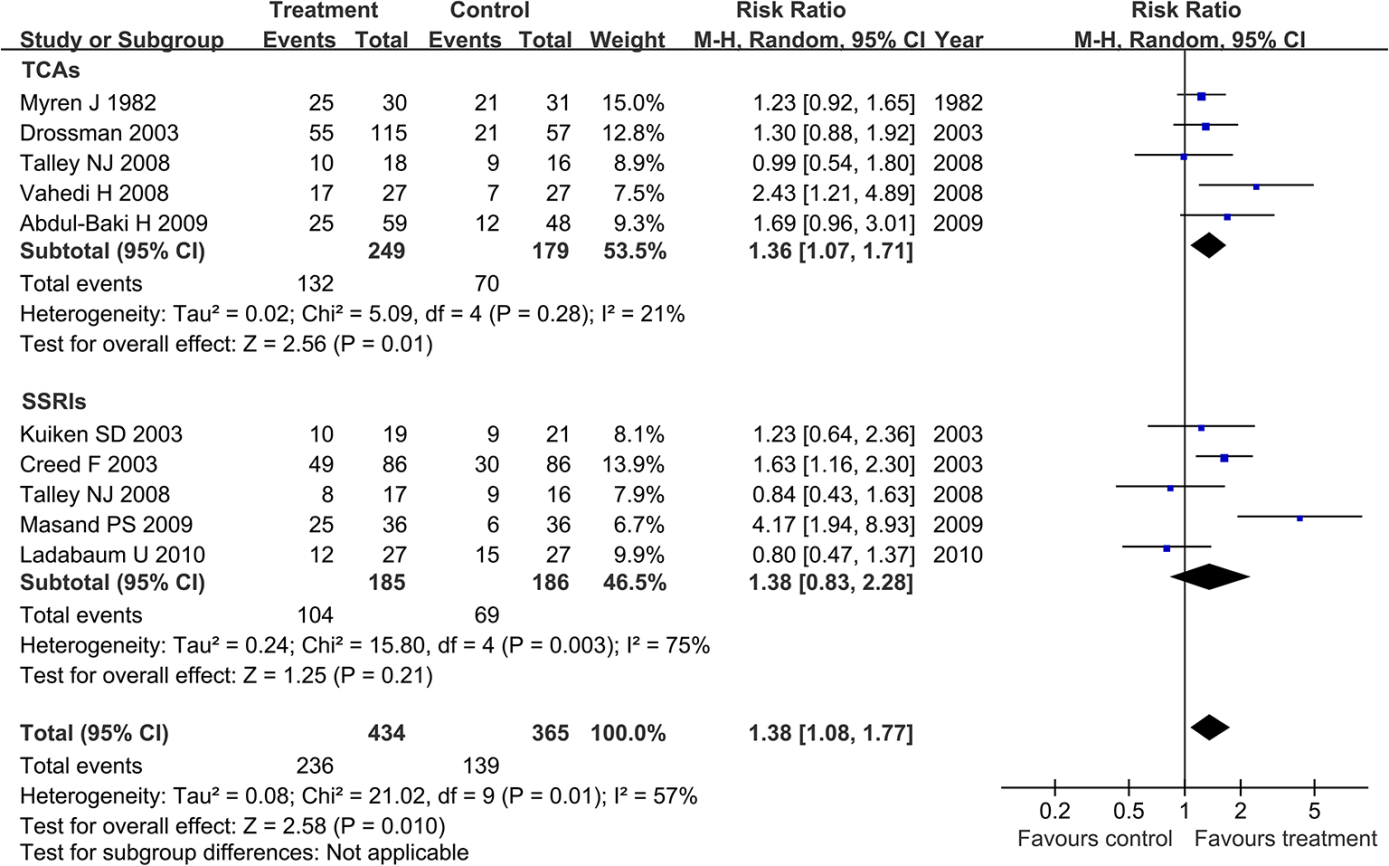
Forest plot of the effects of antidepressants on global symptom relief. Nine articles were included. The random effect model (Mantel-Haenszel method) was applied. Abbreviation: CI confidence interval.

There were 799 IBS patients included in this analysis. The comparison had statistically significant heterogeneity (I^2^ = 57%, P = 0.01). A random effect model was applied to pool effect size. The pooled RR showed treatment with antidepressants can improve global symptoms (RR = 1.38, 95% CI 1.08, 1.77). In the subgroup analysis, treatment with TCAs showed an improvement in global symptoms (RR = 1.36, 95% CI 1.07, 1.71), while treatment with SSRIs showed no statistically significant difference in global symptoms when compared with the control groups (RR = 1.38, 95% CI 0.83, 2.28; Fig 2).

**Fig 3:**
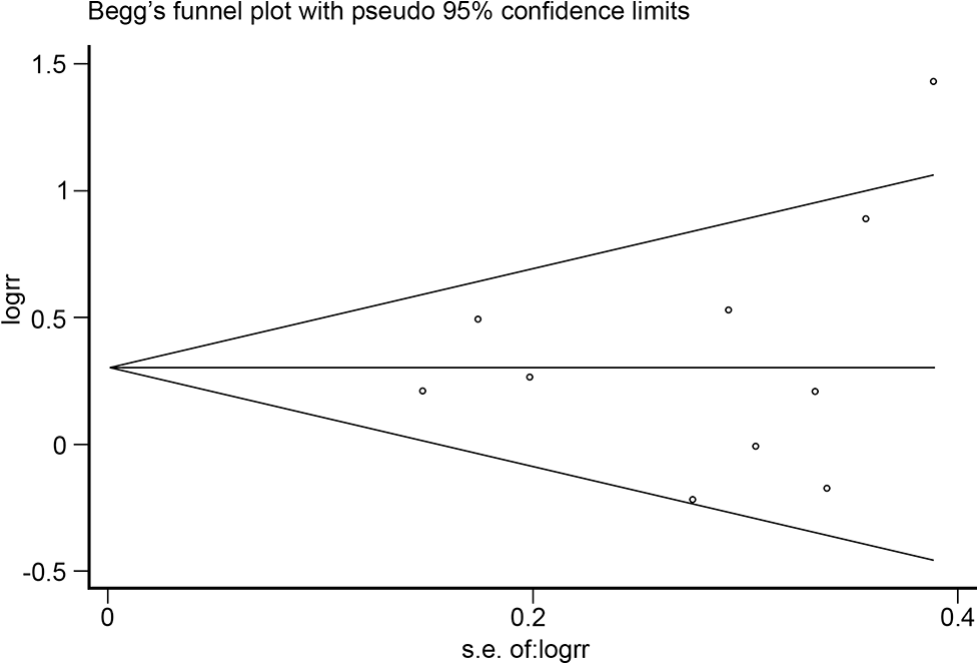
Funnel plot analysis of potential publication bias (for the nine articles seen in Fig 2). The funnel plot was symmetrical and the P-value calculated by Begg’s test was 0.474.

The funnel plot was symmetrical as seen in Fig 3 and the P-value calculated by Begg’s test was 0.474. There was no publication bias among the nine articles.

**Fig 4:**
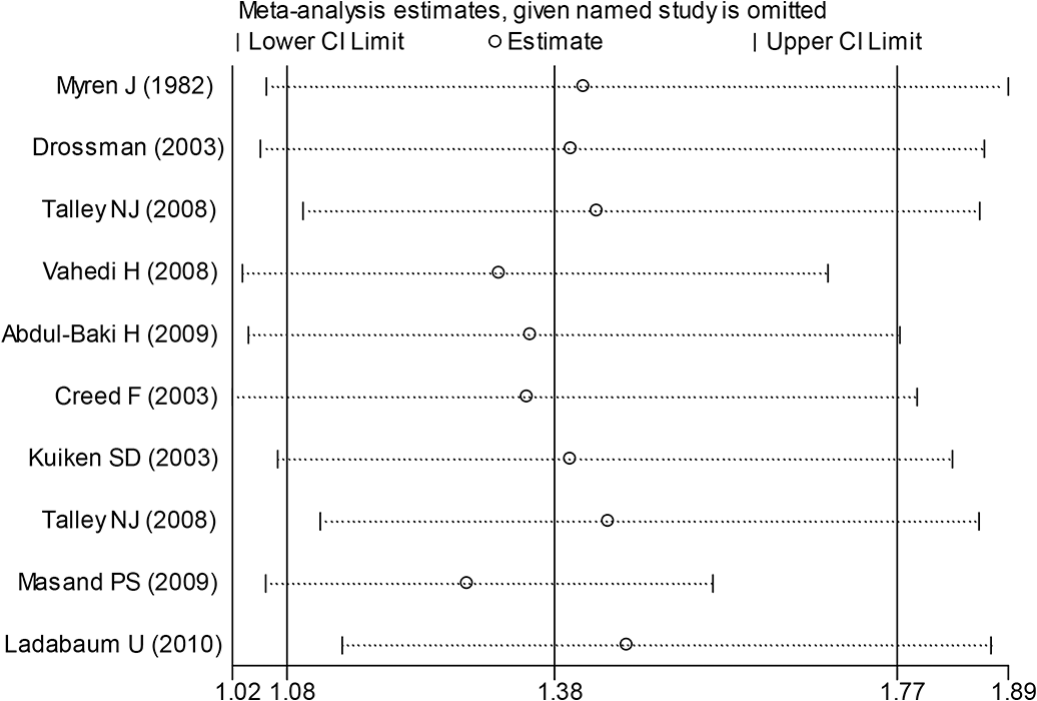
Sensitivity analysis of the effects of antidepressants on global symptom relief.

Subgroup analysis was conducted as the comparison had statistically significant heterogeneity. Through subgroup analysis the heterogeneity was more obvious in the SSRIs group (I^2^ = 75%). In order to find the source of heterogeneity and to judge the robustness of the results, sensitivity analysis was then conducted. From this method, the Masand PS (2009) [14] study showed a marked impact on heterogeneity (Fig 4). Masand PS’s study included 72 IBS patients and the Jadad score was 4. Masand PS indicated that paroxetine had a statistically significant benefit on improving clinical global impression, but it was not effective at reducing composite pain [14].

**Fig 5:**
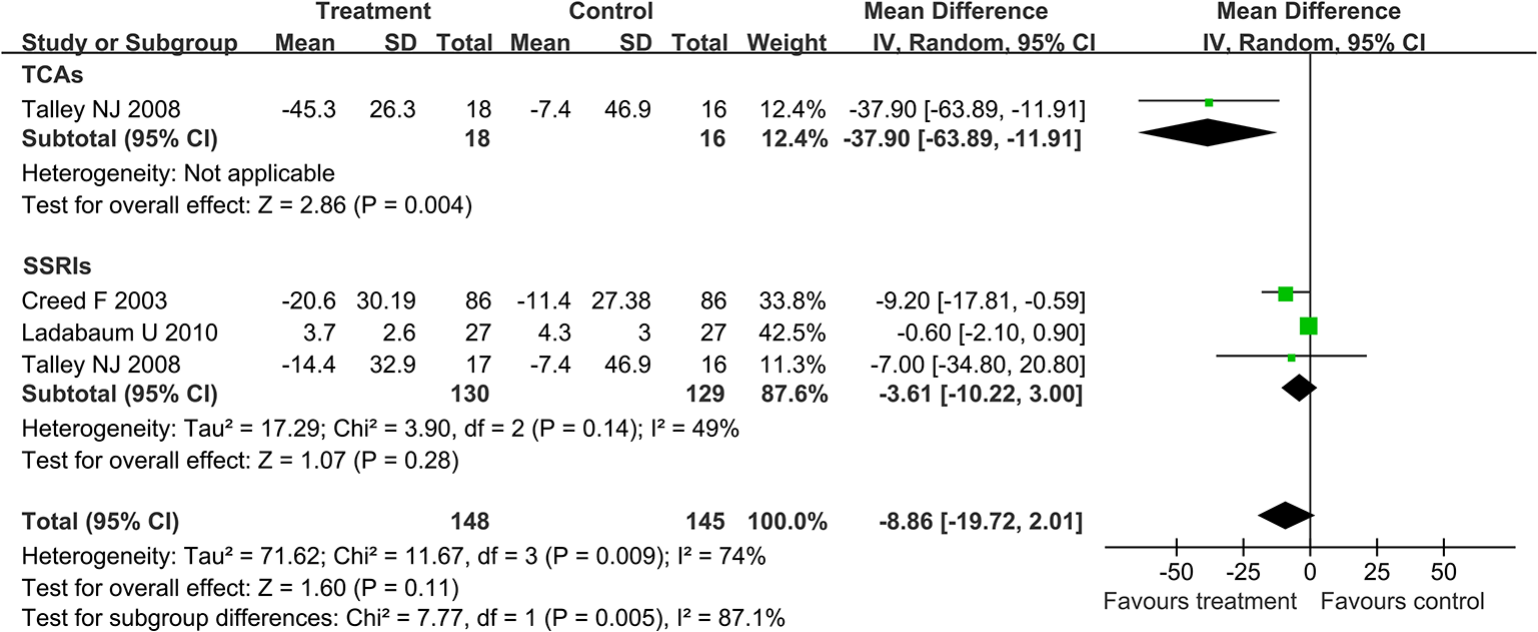
Forest plot of the improvement in the degree of abdominal pain. Three articles were included. The random effect model (Mantel-Haenszel method) was applied. Abbreviation: CI confidence interval.

**Fig 6:**
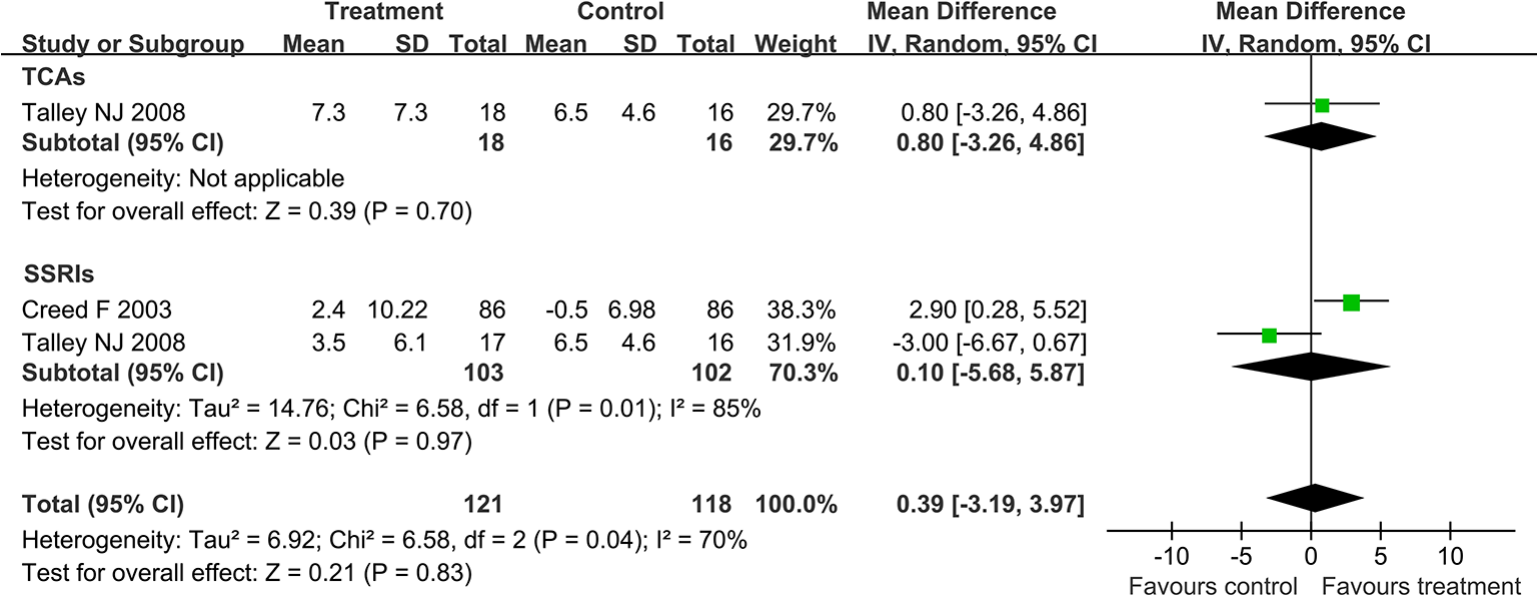
Forest plot of the improvement in quality of life. Two articles were included. The random effect model (Mantel-Haenszel method) was applied. Abbreviation: CI confidence interval.

In order to accurately analyze the effect of antidepressants in IBS, further analysis was conducted on the improvement in the degree of abdominal pain and improvement in quality of life. A random effect model (I^2^ = 74%) was performed on the three articles [15, 18, 20] which studied the effect of antidepressants on abdominal pain and the result was as follows: mean difference (MD) -8.86 with 95% CI (-19.72, 2.01). Subgroup analysis showed no statistically significant benefit of SSRIs (MD -3.61; 95% CI -10.22, 3.00; Fig 5). By performing the random effects model (I^2^ = 70%) on the two articles [15, 20] which studied the effect of antidepressants on quality of life, we found the following result: MD 0.39 with 95% CI (-3.19, 3.97). Subgroup analysis showed no statistically significant benefit of SSRIs (MD 0.1; 95% CI -5.68, 5.87; Fig 6).

**Fig 7:**
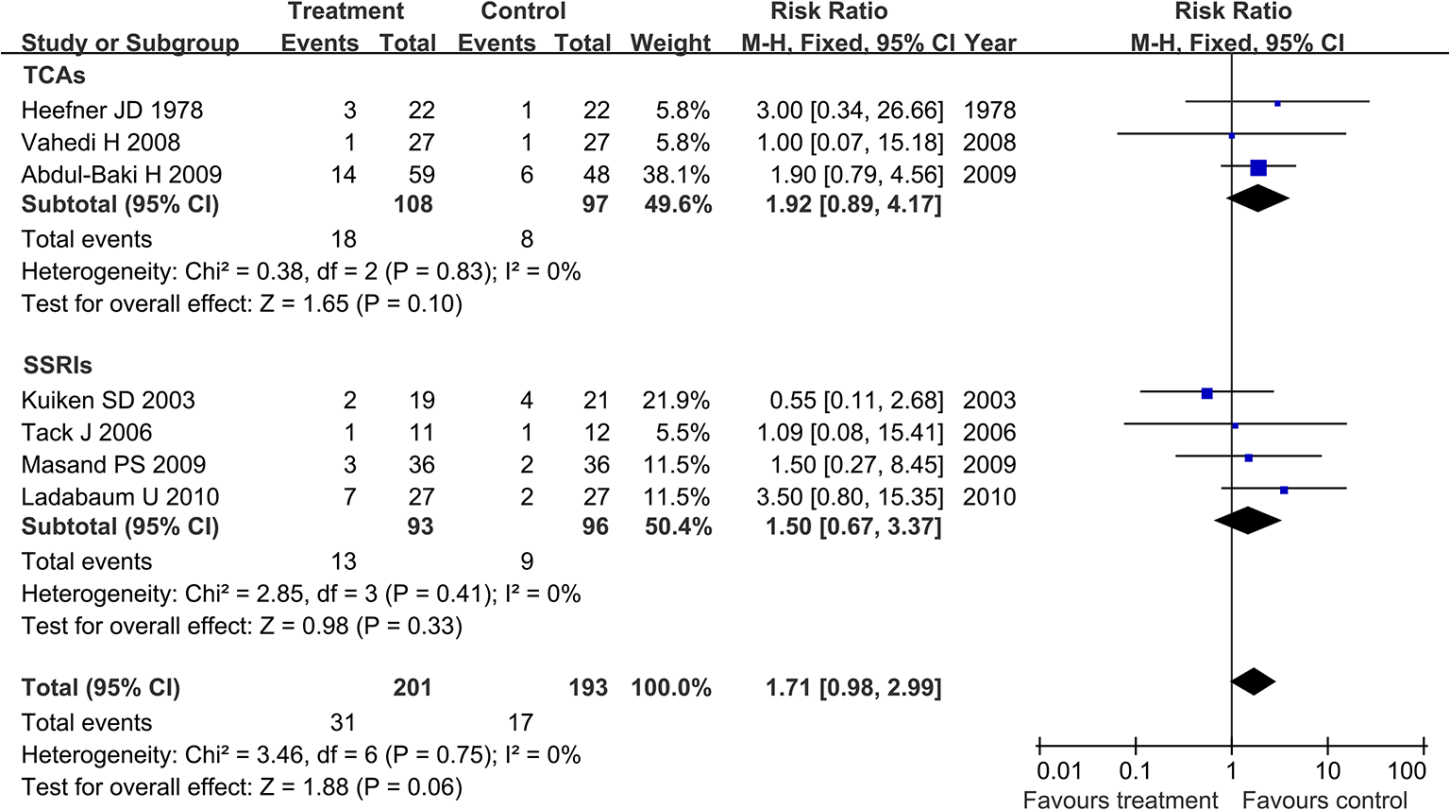
Forest plot of the rate of dropout due to side effects. **Seven articles were included.** The fixed effect model (Mantel-Haenszel method) was applied. Abbreviation: CI confidence interval.

Among the included studies, seven articles evaluated the rate of dropout due to side effects, including three studies [9, 12, 13] for TCAs and four studies [14, 16–18] for SSRIs. In this analysis 394 IBS patients were included. The comparison result showed no statistically significant heterogeneity (I^2^ = 0%, P = 0.75). Therefore, a fixed effect model was applied to pool effect size. The pooled RR of the rate of dropout was 1.71 with 95% CI (0.98, 2.99). The subgroup analysis showed that the pooled RR of the rate of dropout in the TCAs group was 1.92 with 95% CI (0.89, 4.17). In the SSRIs group, the pooled RR of the rate of dropout was 1.5 with 95% CI (0.67, 3.37; Fig 7).

**Fig 8:**
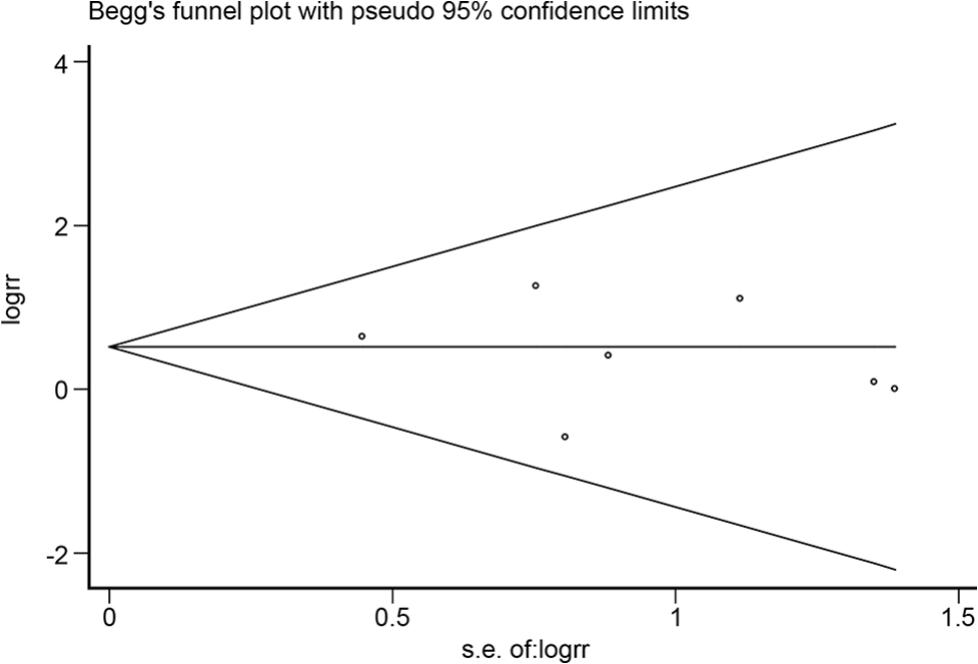
Funnel plot analysis of potential publication bias (for the seven articles seen in Fig 7). The funnel plot was symmetrical, as seen in Fig 8, and the P-value calculated by Begg’s test was 0.368.

The funnel plot was symmetrical as seen in Fig 8 and the P-value calculated by Begg’s test was 0.368. It was concluded that there was no publication bias among these seven articles.

**Fig 9:**
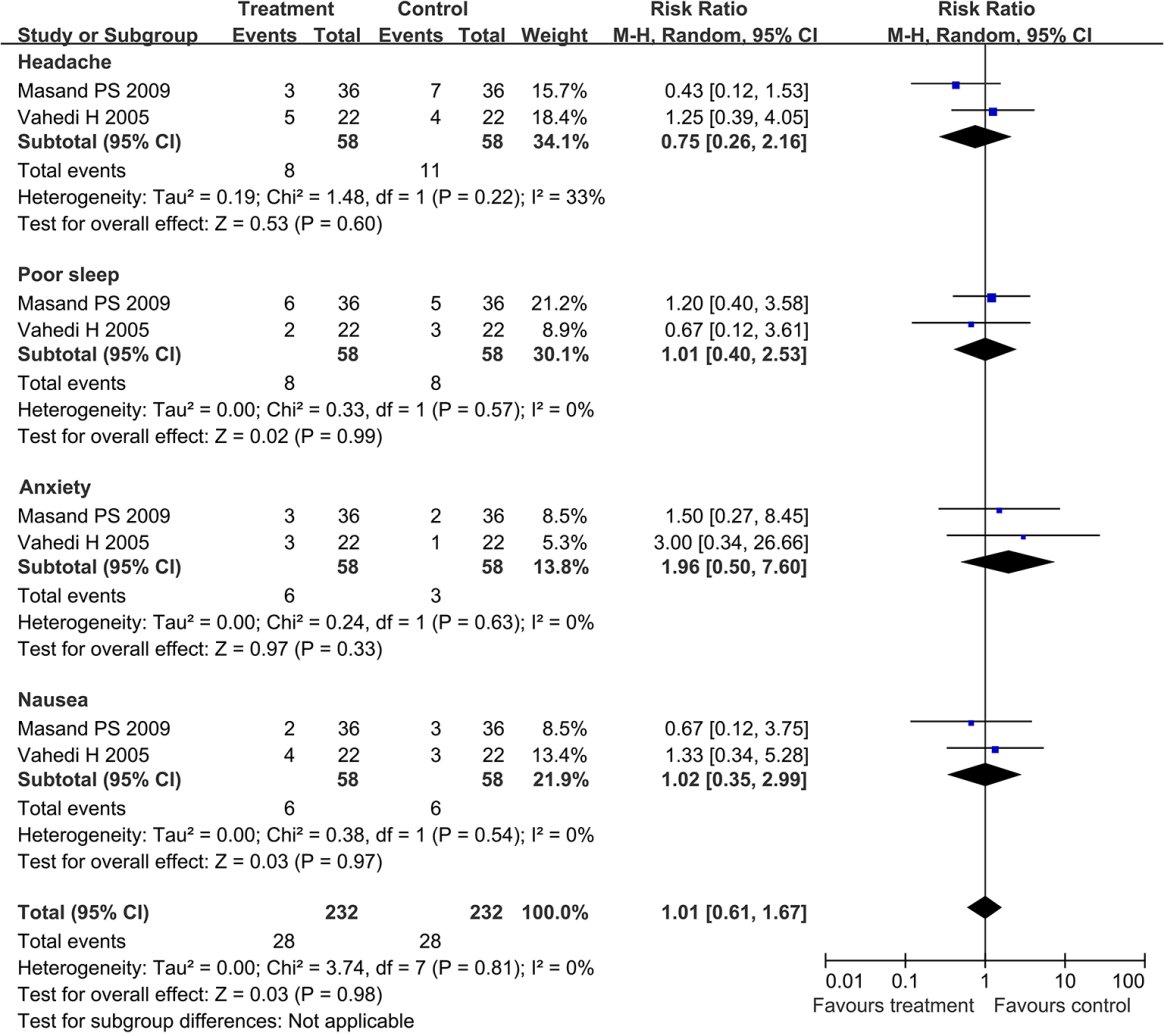
Forest plot of the rate of four types of adverse events in SSRIs group. Two articles were included. The random effect model (Mantel-Haenszel method) was applied. Abbreviation: CI confidence interval.

In the SSRIs group, there were two articles [14, 19] providing four types of adverse events both in the treatment groups and control groups. The pooled RR of experiencing headache, poor sleep, anxiety, and nausea was 0.75 with 95% CI (0.26, 2.16), 1.01 with 95% CI (0.40, 2.53), 1.96 with 95% CI (0.50, 7.60), and 1.02 with 95% CI (0.35, 2.99), respectively (Fig 9).

## Discussion

This meta-analysis indicated that TCAs could improve global symptoms, but SSRIs showed no statistically significant difference when compared with the control groups. The results were consistent with a systematic review that was recently published by Bundeff and Woodis [21] who believed the data evaluating the use of SSRIs in the treatment of IBS were conflicting. They advised that SSRIs should not be routinely used to treat IBS in those patients without comorbid psychiatric conditions [21].

During the subgroup analysis, statistically significant heterogeneity was present in the SSRIs group. The SSRIs group contained five studies. Two studies analyzed the effect of paroxetine and indicated that paroxetine can improve global symptoms [14, 15]. Two other studies using citalopram and indicated that citalopram had no statistically significant differences when compared with the control groups [18, 20]. The fifth study explored the effect of fluoxetine, but did not find that it had any benefit in improving global symptom comparing to the placebo [16]. The above analysis suggests that paroxetine has more obvious beneficial effects. However, these two studies had significant levels of heterogeneity [14, 15]. After the removal of these two studies, the level of heterogeneity was significantly decreased and the I^2^ fell to zero. The Jadad scores of the two studies were lower than those of the other three studies. Although one of the studies contained a sample set that was larger than the others, the subjects selected in that study were IBS patients with ‘severe’ symptoms who had failed to respond to conventional medical treatments; furthermore, the control group in that study had been given conventional therapy rather than placebo. Therefore, this was not a double-blind study and the antidepressants might have acted through a placebo effect [15]. The other study was described as a double-blind, randomized, placebo-controlled trial, but the method of randomization and concealment of allocation were not stated [14]. As a result of apparent heterogeneity across these two studies, the effect of paroxetine should be considered with some caution.

The assessments of the effects of antidepressants on abdominal pain and quality of life were relative common measurements. However, the assessment criteria used in different studies were inconsistent. Hence, only a limited number of studies evaluating both symptoms were included according to the strict criteria of this meta-analysis. The paucity of data might not have had adequate power to detect significant differences, which may explain TCAs showed no significant benefit on quality of life but could improve global symptomatology. While abdominal pain is a main symptom of IBS, abdominal discomfort, the sense of bloating and change in bowel habit are all likely to affect the overall experience of patients. Thus the evaluation of global symptom improvements could cover interferences that result from all symptoms of IBS and it was more representative.

Some studies have reported the effect of TCAs may be enhanced in IBS with diarrhea, while the effect of SSRIs may be improved in IBS with constipation [22]. In this meta-analysis, most studies did not differentiate between the subtypes of IBS. Therefore, it was not feasible to assess the different antidepressants in the treatment of different subtypes of IBS. In the TCAs group, Vahedi et al. only included patients having IBS with diarrhea. Vahedi et al. found that amitriptyline was significantly more effective than placebo in decreasing the incidence of loose stools and the feeling of incomplete defecation [12]. In the SSRIs group, Vahedi et al. only included patients having IBS with constipation and found that fluoxetine showed greater reduction in the feeling of abdominal discomfort and bloating, greater increase in frequency of bowel movements, and a decrease in the consistency of stools [19].

IBS patients often have more psychological disorders than healthy people. However, most of the studies did not include patients with clinical psychological comorbidity. Therefore, it is hard to determine whether psychological comorbidity influences the effects of antidepressants. It is also unclear whether IBS symptoms are relieved by the improvement of psychological symptoms. In the TCAs group, Abdul-Baki et al. excluded patients with clinical depression, and found imipramine may be useful in treating IBS related symptoms [13]. In the SSRIs group, three studies excluded patients with psychological comorbidity and the result was controversial. Ladabaum et al. reported that citalopram and Kuiken et al. found that fluoxetine were not superior to placebo in treating non-depressed IBS patients [16, 18]. However, Tack et al. reported that citalopram significantly improved IBS symptoms and the therapeutic effect was independent of the effects on anxiety or depression [17].

The duration of included studies was between one and three months. Few articles with long-term follow-up have been published. In 2003, Creed et al. reported a RCT comparing psychotherapy, antidepressants and usual therapy [15]. After 15-month follow-up, in all three groups, there was no significant difference in the severity and frequency of abdominal pain. However, both antidepressant and psychotherapy groups showed greater improvements over usual treatments in SF-36 health related quality of life assessments [15].

Antidepressants were considered to be a safe treatment for IBS. Few serious adverse events were reported in the placebo-controlled studies. In the TCAs group, the most common side effects were dry mouth, drowsiness, and palpitations. In the SSRIs group, the adverse events often reported were poor sleep, headache, anxiety, and nausea. For the outcome of dropout because of side effects, it was found that the pooled RR showed no statistically significant difference between the treatment and control groups. The rate of common adverse events of SSRIs also showed no statistically significant difference between the treatment and control groups. Based on the above analysis, it is safe to use antidepressants to treat IBS.

The strengths of our study were as follows: (1) most of included trials were of high quality (Jadad score ≥4); (2) The effects of antidepressants were evaluated by global symptom improvement. With this method, we are able to assess the effects of antidepressants on the global symptoms rather than limiting the symptom profile to abdominal pain. Besides, these fixed criteria avoid the random assessment of relief in abdominal pain or the improvement in global symptoms at the same time and therefore avoid the overestimation of the effects of antidepressants; (3) previously published meta-analysis rarely referred to adverse events. In our meta-analysis, we used the dropout rate because of side effects to evaluate the safety of antidepressants and we analyzed the pooled rate of some common side effects. This meta-analysis also has some shortcomings. Firstly, as a result of the apparent heterogeneity across studies and a paucity of included studies, the interpretation of the effects of SSRIs requires caution. Secondly, because the assessment of improvements in each study was different and not detailed, it was difficult to accurately assess the effects of antidepressants. Thirdly, language restrictions may have neglected some reports that were not published in English.

## Conclusions

TCAs can improve the global symptoms of irritable bowel syndrome, while there was no strong evidence to confirm the effectiveness of SSRIs in the treatment of IBS. Large and long-term RCTs are needed to assess the efficacy of SSRIs for this purpose. Although it is not recommended to use SSRIs to treat IBS routinely based on the current evidence, it is a safe practice and does not increase the risk of side effects. Therefore, after an individualized assessment, clinicians are encouraged to use antidepressants in scenarios where patients have failed to respond to conventional therapies for IBS and also have psychiatric comorbidities.

## Supporting Information

S1 PRISMA Checklist PRISMA Checklist. (DOC)
