# Supplementary material for: Efficacy and Safety of Antidepressants for the Treatment of Irritable Bowel Syndrome: A Meta-Analysis
Source: PLoS One. 2015 Aug 7;10(8):e0127815. doi: 10.1371/journal.pone.0127815 (PMC4529302; doi:10.1371/journal.pone.0127815)
Supplement: S1 PRISMA Checklist — (DOC) [file pone.0127815.s001.doc]

| **Section/topic** | **#** | **Checklist item** | **Reported on page #** |
| --- | --- | --- | --- |
| **TITLE** | | |  |
| Title | 1 | “Efficacy and Safety of Antidepressants for the Treatment of Irritable Bowel Syndrome: A Meta-Analysis” | 1 |
| **ABSTRACT** | | |  |
| Structured summary | 2 | “Abstract” | 2-3 |
| **INTRODUCTION** | | |  |
| Rationale | 3 | “Introduction” | 4-5 |
| Objectives | 4 | “Introduction” | 4-5 |
| **METHODS** | | |  |
| Protocol and registration | 5 | This meta-analysis was not registered. | N/A |
| Eligibility criteria | 6 | “inclusion and exclusion criteria” | 5-6 |
| Information sources | 7 | “Search strategy” | 5 |
| Search | 8 | “Search strategy” | 5 |
| Study selection | 9 | “Search strategy”, “inclusion and exclusion criteria” and figure 1 | 5-6\ Figure 1 |
| Data collection process | 10 | “Outcomes”, “Literature quality evaluation“, “Data extraction and analysis”, table1 and table 2 | 6-8\ table1 \table 2 |
| Data items | 11 | “Outcomes”, “Literature quality evaluation“, “Data extraction and analysis”, table1 and table 2 | 6-8\ table1 \table 2 |
| Risk of bias in individual studies | 12 | “Literature quality evaluation“ and “Data extraction and analysis” | 6-8 |
| Summary measures | 13 | “Outcomes”, “Literature quality evaluation“, “Data extraction and analysis”, table1 and table 2 | 6-8\ table1 \table 2 |
| Synthesis of results | 14 | “Data extraction and analysis” | 7-8 |

Page 1 of 2

| **Section/topic** | **#** | **Checklist item** | **Reported on page #** |
| --- | --- | --- | --- |
| Risk of bias across studies | 15 | “Literature quality evaluation“ and “Data extraction and analysis” | 6-8 |
| Additional analyses | 16 | “Data extraction and analysis” | 7 |
| **RESULTS** | | |  |
| Study selection | 17 | “Results” and figure 1 | 8\ figure 1 |
| Study characteristics | 18 | “Results”, table1, 2 | 8\ table1, 2 |
| Risk of bias within studies | 19 | “Results”, figure 3, 8 | 9-10\ figure 3, 8 |
| Results of individual studies | 20 | “Results”, table1, 2, figure 2, 5, 6, 7, 9 | 8-10\table1, 2, figure 2, 5, 6, 7, 9 |
| Synthesis of results | 21 | “Results”, table1, 2, figure 2, 5, 6, 7, 9 | 8-10\table1, 2, figure 2, 5, 6, 7, 9 |
| Risk of bias across studies | 22 | “Literature quality evaluation“ and “Data extraction and analysis” | 6-8 |
| Additional analysis | 23 | “Results”, figure 2, 4, 5, 6, 7, 9 | 9-10\ figure 2, 4, 5, 6, 7, 9 |
| **DISCUSSION** | | |  |
| Summary of evidence | 24 | Discussion | 10-14 |
| Limitations | 25 | Discussion | 14-15 |
| Conclusions | 26 | Conclusions | 15 |
| **FUNDING** | | |  |
| Funding | 27 | No funding for our meta-analysis. | N/A |

*From:*  Moher D, Liberati A, Tetzlaff J, Altman DG, The PRISMA Group (2009). Preferred Reporting Items for Systematic Reviews and Meta-Analyses: The PRISMA Statement. PLoS Med 6(6): e1000097. doi:10.1371/journal.pmed1000097

For more information, visit: **www.prisma-statement.org**.

Page 2 of 2
